# Supplementary material for: Dissociation protocols used for sarcoma tissues bias the transcriptome observed in single-cell and single-nucleus RNA sequencing
Source: BMC Cancer. 2023 May 31;23:488. doi: 10.1186/s12885-023-10977-1 (PMC10230784; doi:10.1186/s12885-023-10977-1)
Supplement: Supplementary file 1 — Additional file 1: Figure S1. Gating strategy to enrich for single-cells and single-nuclei. Figure S2. Consensus of DEGs between Sarcoma Subtypes when comparing Warm and Nuclei Protocols. Figure S3. DEGs Biases introduced by Warm and Cold Protocols. Figure S4. Differences in Sequencing Performance. Figure S5. Classifying sample type based on bias scores for ES. Figure S6. Classifying sample type based on bias scores for OS and DSRCT. Figure S7. Classifying sample type based on bias scores for Neuroblastoma. Figure S8. Biases associated gene length in gene signatures. Figure S9. Integration recovers matching cell states from different dissociation methods for ES. Figure S10. Integration recovers matching cell states from different dissociation methods for DSRCT. Table S1. Number of cells analyzed pre- and post-quality control. Table S2. Gene sets curated from literature analyzed in PDX samples. The AddModuleScore function in Seurat v3 was used to observe the averaged gene expression of the gene sets. Table S3. Differentially expressed genes between Warm and Nuclei protocols for ES. Wilcoxon test was used to compare gene expression between protocols. Table S4. Differentially expressed genes between Warm and Nuclei protocols for OS. Wilcoxon test was used to compare gene expression between protocols. Table S5. Differentially expressed genes between Warm and Nuclei protocols for DSRCT. Wilcoxon test was used to compare gene expression between protocols. Table S6. Differentially expressed genes between Warm and Cold protocols for DSRCT. Wilcoxon test was used to compare gene expression between protocols. Table S7. Differentially expressed genes between Warm and Cold protocols for ES. Wilcoxon test was used to compare gene expression between protocols. Table S8. Top 200 longest genes with the most polyA regions. The polyA stretches were defined as greater than 15 A repeats within the full-length cDNA, including intronic and exonic regions for every gene. [file 12885_2023_10977_MOESM1_ESM.zip › Supplementary Materials_BMCCancer_RevisionR1.docx]

**Additional Figures
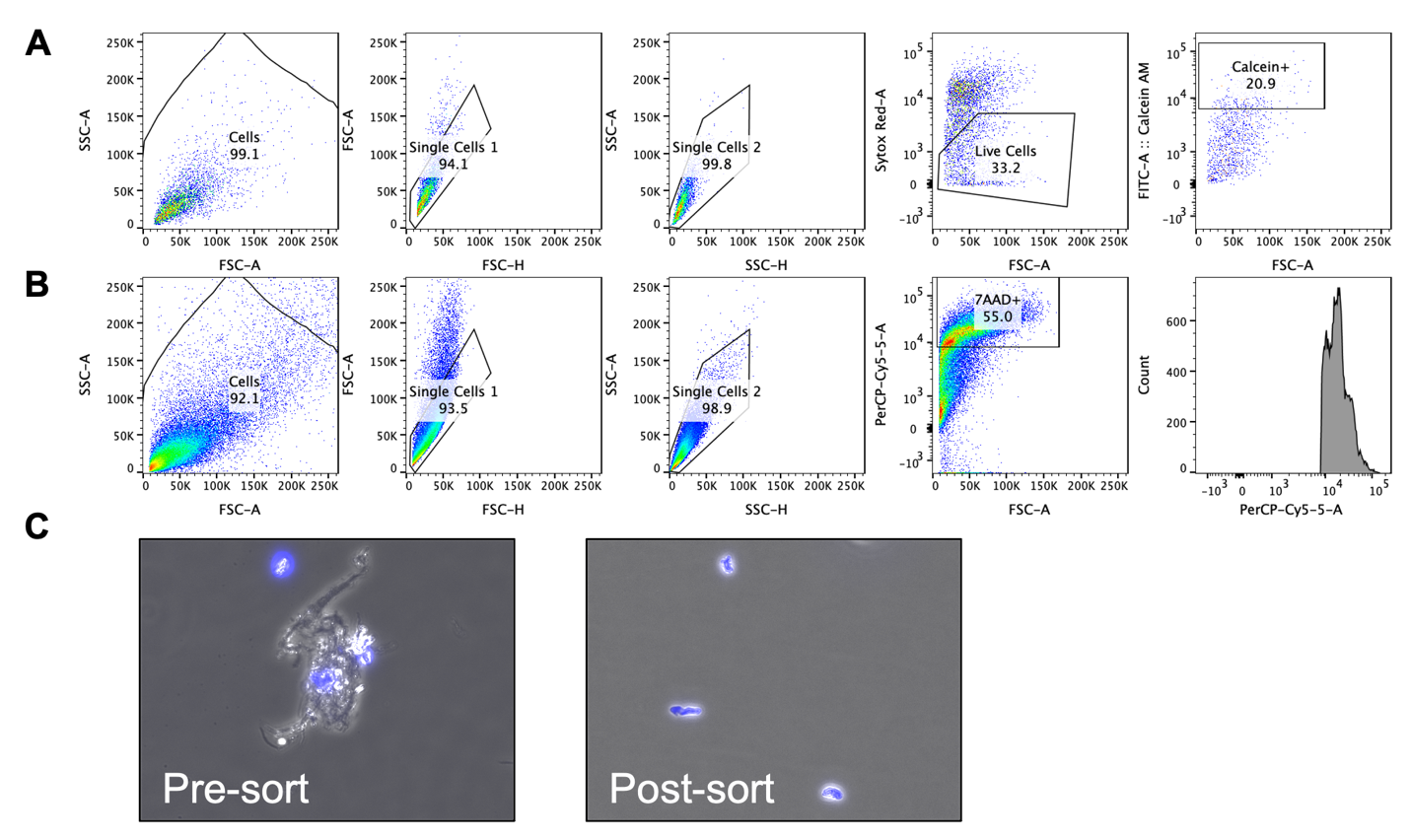
**

**Figure S1. Gating strategy to enrich for single-cells and single-nuclei. A** Cells are gated by SSC-A and FSC-A (first plot). Doublets are removed first by FSC-A and FSC-H and then by SSC-A and SSC-H (second and third plots). Live cells are gated from dead cells by Sytox Red-A (dead cells) and FSC-A (fourth plot). Live cells are sorted using FITC-A Calcein-AM (fifth plot). **B** Nuclei are gated by SSC-A and FSC-A (first plot). Doublets are removed first by FSC-A and FSC-H and then by SSC-A and SSC-H (second and third plots). Nuclei are gated from debris and intact cells by PerCP-Cy5-5-A (7-AAD, fourth plot). The histogram shows DNA content of nuclei (fifth plot). **C** Staining of nuclei with DAPI pre- and post-sort. Single nuclei without debris are enriched with sorting.

**
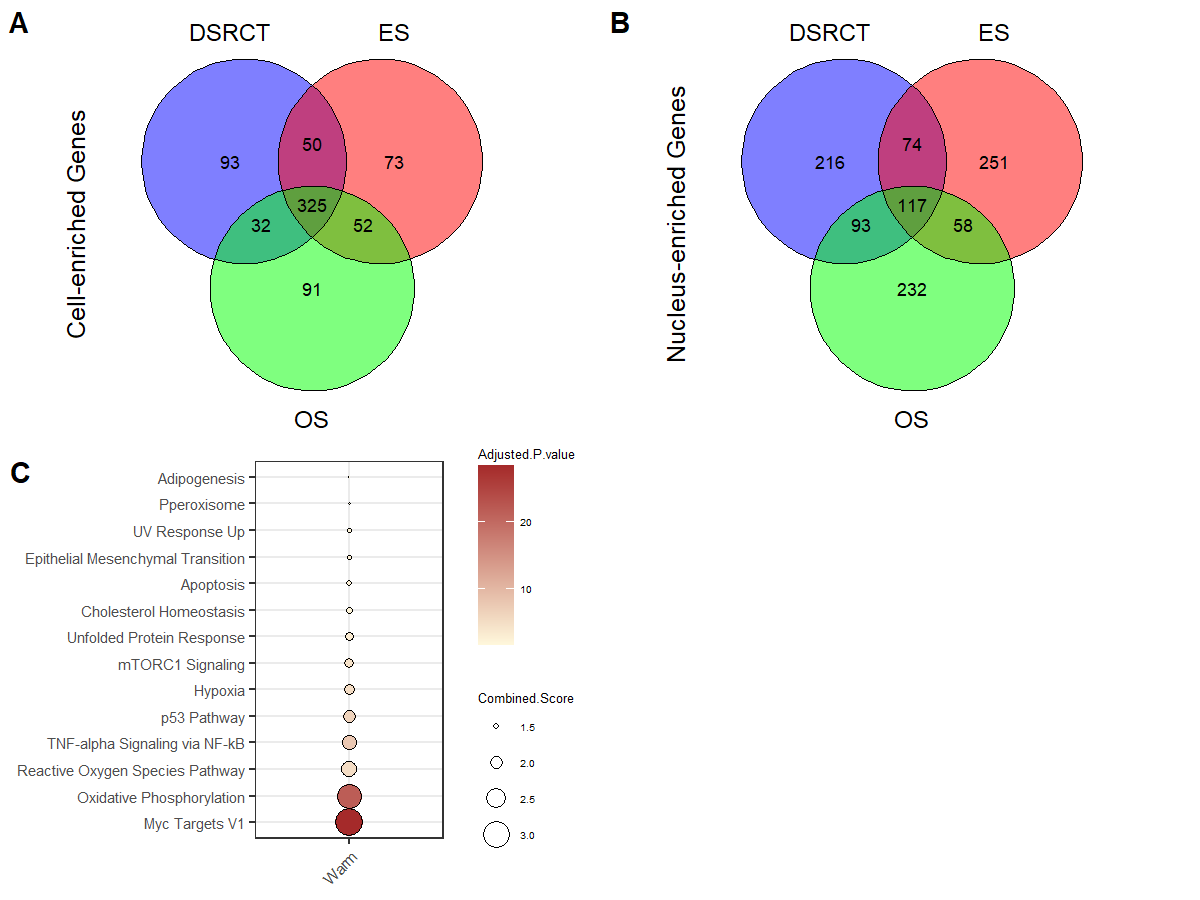
**

**Figure S2. Consensus of DEGs between Sarcoma Subtypes when comparing Warm and Nuclei Protocols. A** Venn diagram of cell-enriched genes with 325 consensus genes enriched in the Warm protocol compared to the Nuclei protocol. **B** Venn diagram of nucleus-enriched genes with 117 consensus genes enriched in the Nuclei protocol compared to the Warm protocol. **C** Dot plot of enrichR scores of the Hallmark gene sets from MSigDB using only consensus genes. Scale reports -log10 Adjusted P-value.

**
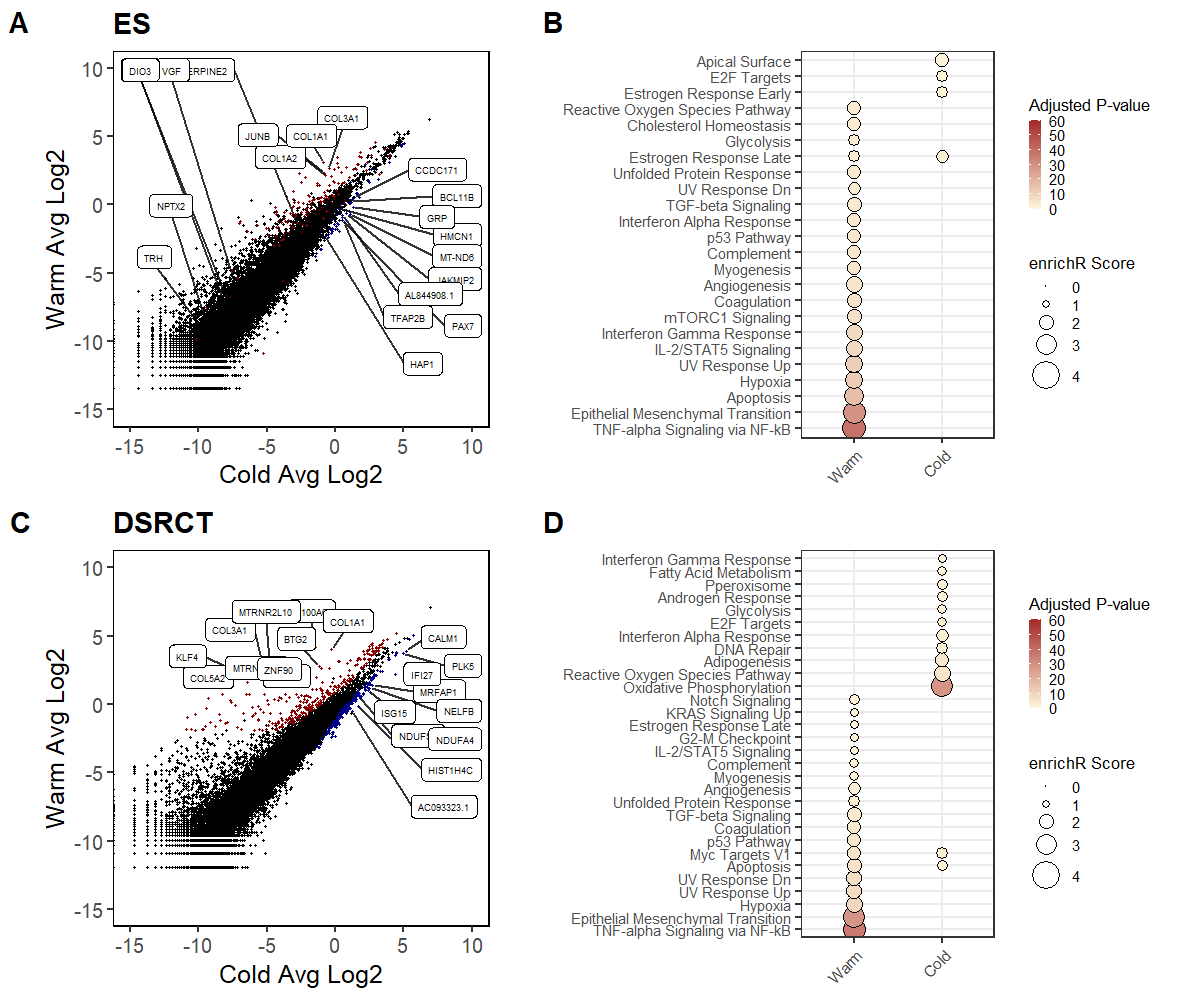
**

**Figure S3.** **DEGs Biases introduced by Warm and Cold Protocols.** Scatter plot of log transformed gene expression levels between Warm and Cold. Red indicates up-regulated in Warm, and blue indicates up-regulated in Cold with a p-value < 0.05. Black is non-significant. Dot plot of enrichR scores of the Hallmark gene sets from MSigDB. Plots are shown for DSRCT **A, B**; and ES **C, D**. Scale reports -log10 Adjusted P-value.

**
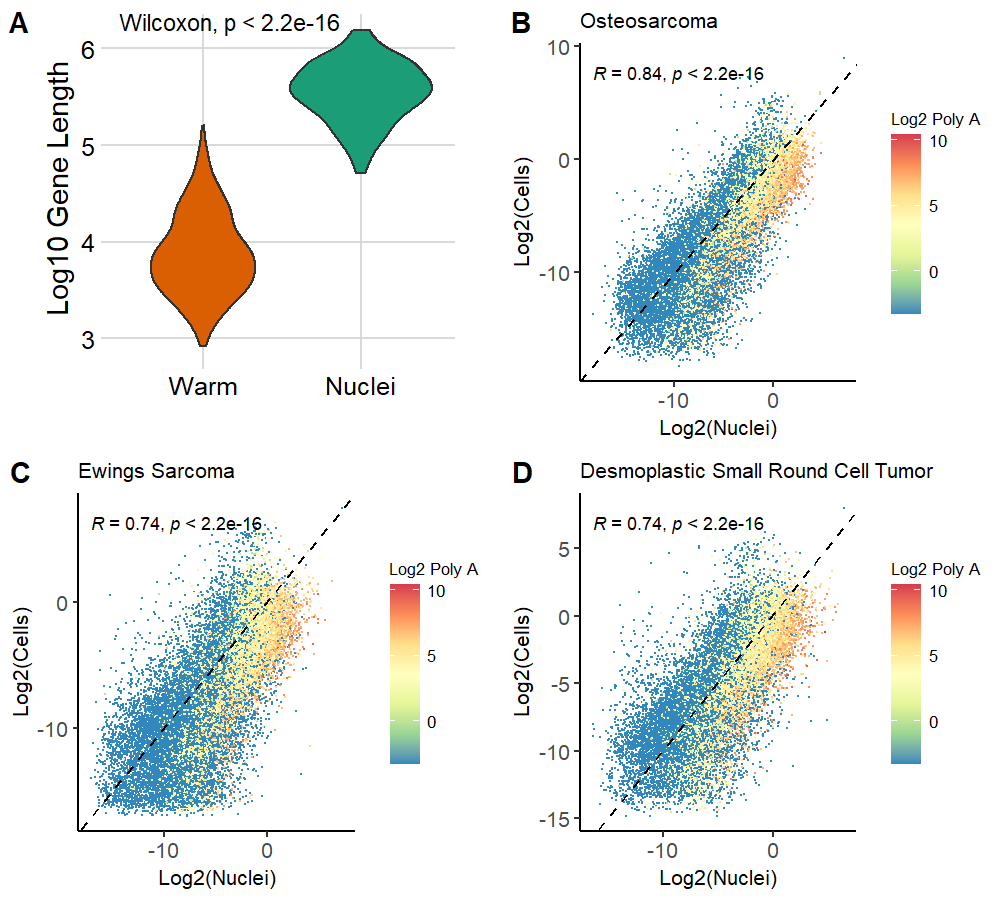
**

**Figure S4. Differences in Sequencing Performance. A** Log-log plot of gene expression between cells and nuclei colored by the number of PolyA regions for **B** OS, **C** ES, and **D** DSRCT compared between Nuclei and Warm protocols.


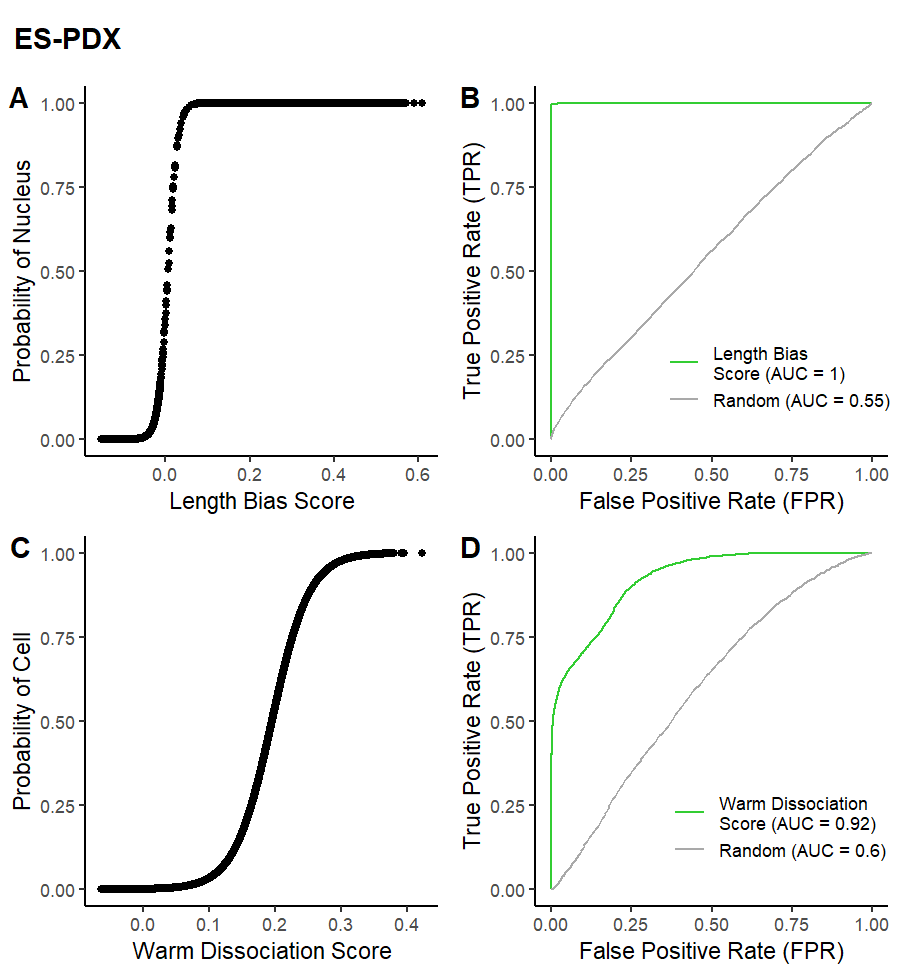


**Figure S5. Classifying sample type based on bias scores for ES.**

**A** Predicting nuclei on test data after training data on Length Bias Score using logistic regression. **B** Receiver operating characteristic (ROC) curve was used to evaluate the performance of the Length Bias Score to classify nuclei. **C** Predicting cells on test data after training data on Warm Dissociation Score using logistic regression. **D** ROC curve was used to evaluate the performance of the Warm Dissociation Score to classify cells.


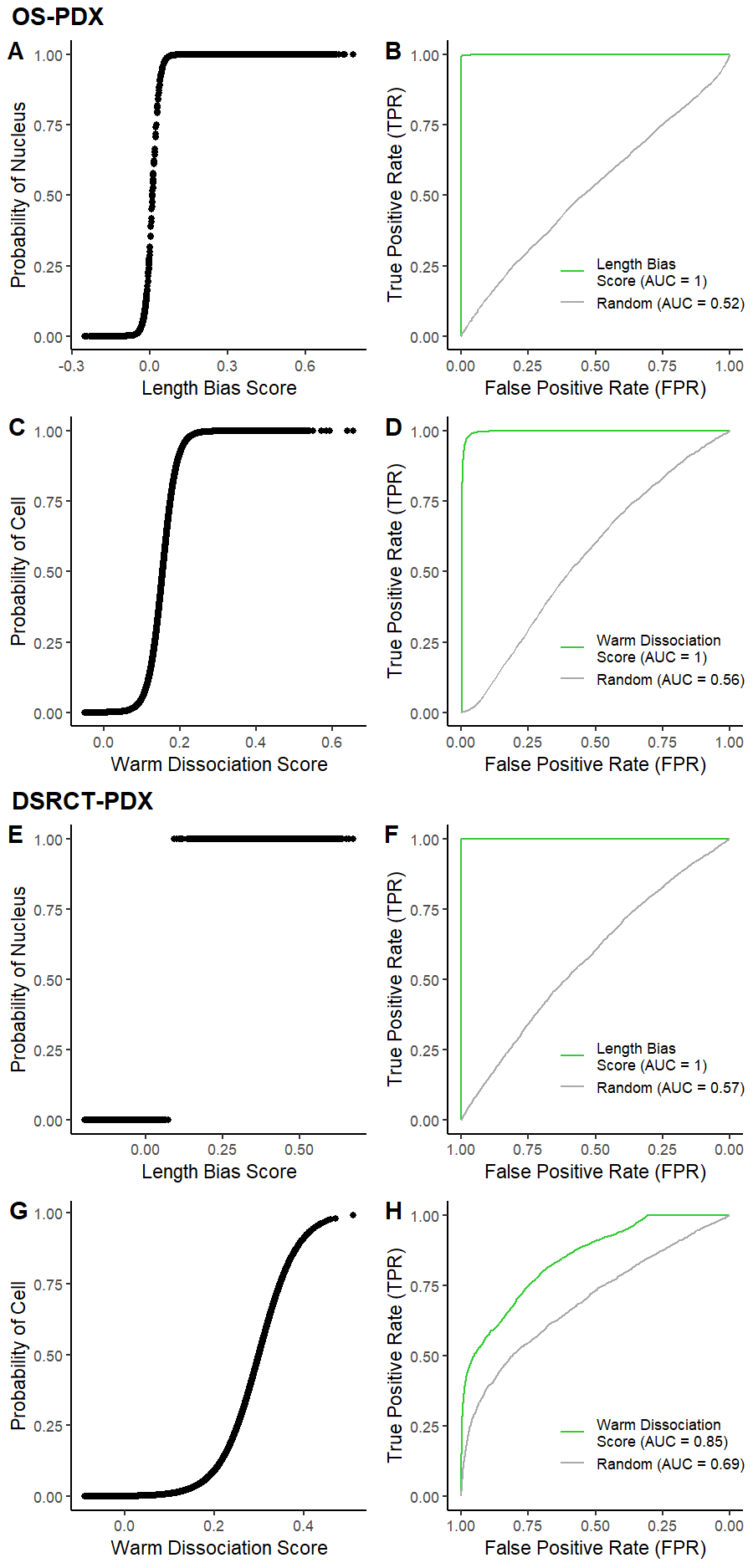


**Figure S6. Classifying sample type based on bias scores for OS and DSRCT. A** Probability of nuclei based on a logistic regression model of Length Bias Score for OS. **B** ROC curve for using Length Bias Score to classify nuclei. **C** Probability of cell based on a logistic regression model of Warm Dissociation Score for OS. **D** ROC curve for using Warm Dissociation Score to classify cells. **E** Probability of nuclei based on a logistic regression model of Length Bias Score for DSRCT. **F** ROC curve for using Length Bias Score to classify nuclei. **G** Probability of cell based on a logistic regression model of Warm Dissociation Score for DSRCT. **H** ROC curve for using Warm Dissociation Score to classify cells.


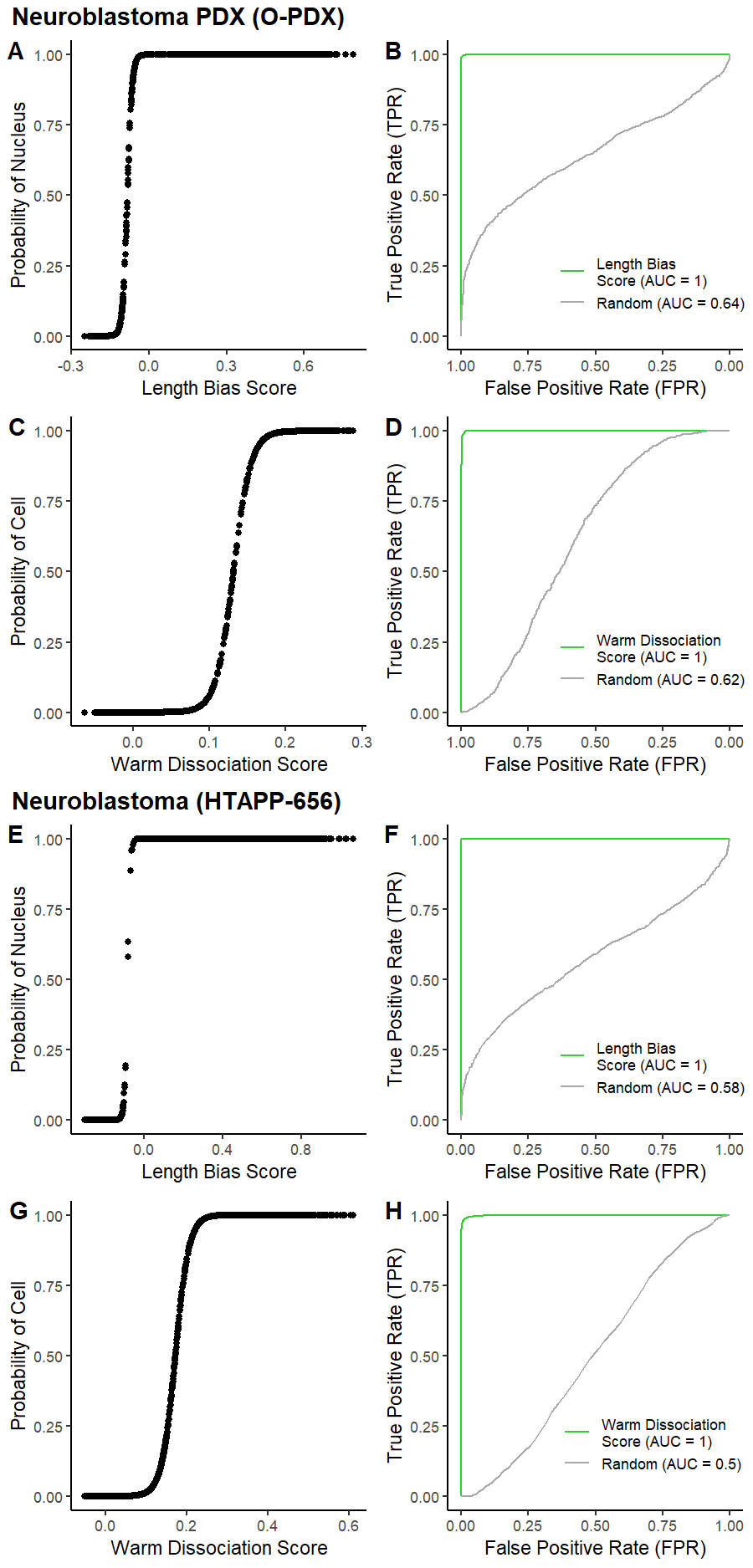


**Figure S7. Classifying sample type based on bias scores for Neuroblastoma. A** Probability of nuclei based on a logistic regression model of Length Bias Score for O-PDX. **B** ROC curve for using Length Bias Score to classify nuclei. **C** Probability of cell based on a logistic regression model of Warm Dissociation Score for O-PDX. **D** ROC curve for using Warm Dissociation Score to classify cells. **E** Probability of nuclei based on a logistic regression model of Length Bias Score for HTAPP-656. **F** ROC curve for using Length Bias Score to classify nuclei. **G** Probability of cell based on a logistic regression model of Warm Dissociation Score for HTAPP-656. **H** ROC curve for using Warm Dissociation Score to classify cells.


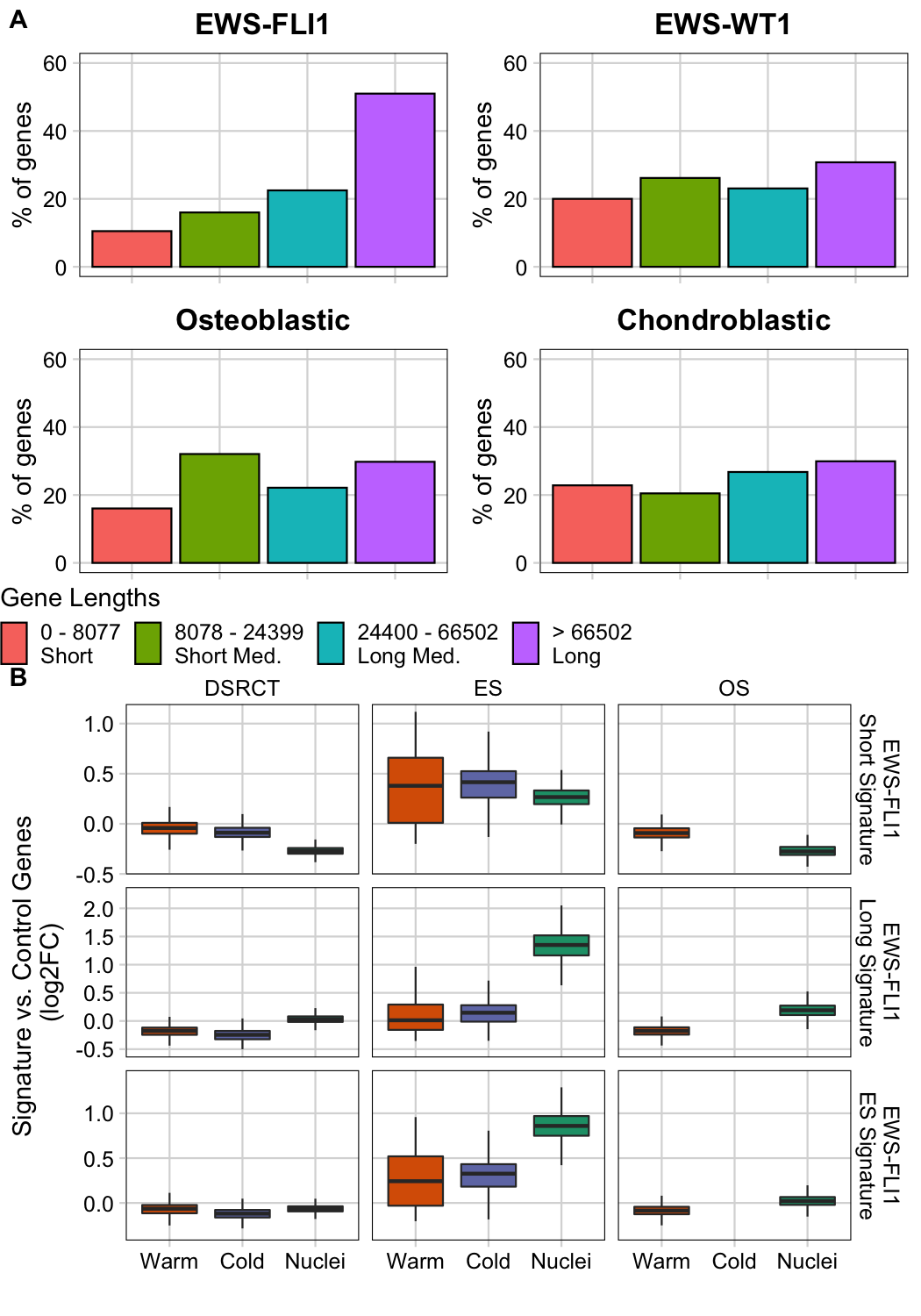


**Figure S8.** **Biases associated gene length in gene signatures.** **A** The gene set in each sarcoma signature was split into four bins of gene length quartiles; Short (0 – 8077 nt), Short Med. (8078 – 24399 nt), Long. Med. (24400 – 66502 nt), and Long (> 66502 nt). **B** EWS-FLI1 ES signature was split by gene length; short < 66502 nt (top row) and long ≥ 66502 nt (middle row). The original gene set without adjustment is in the bottom row.


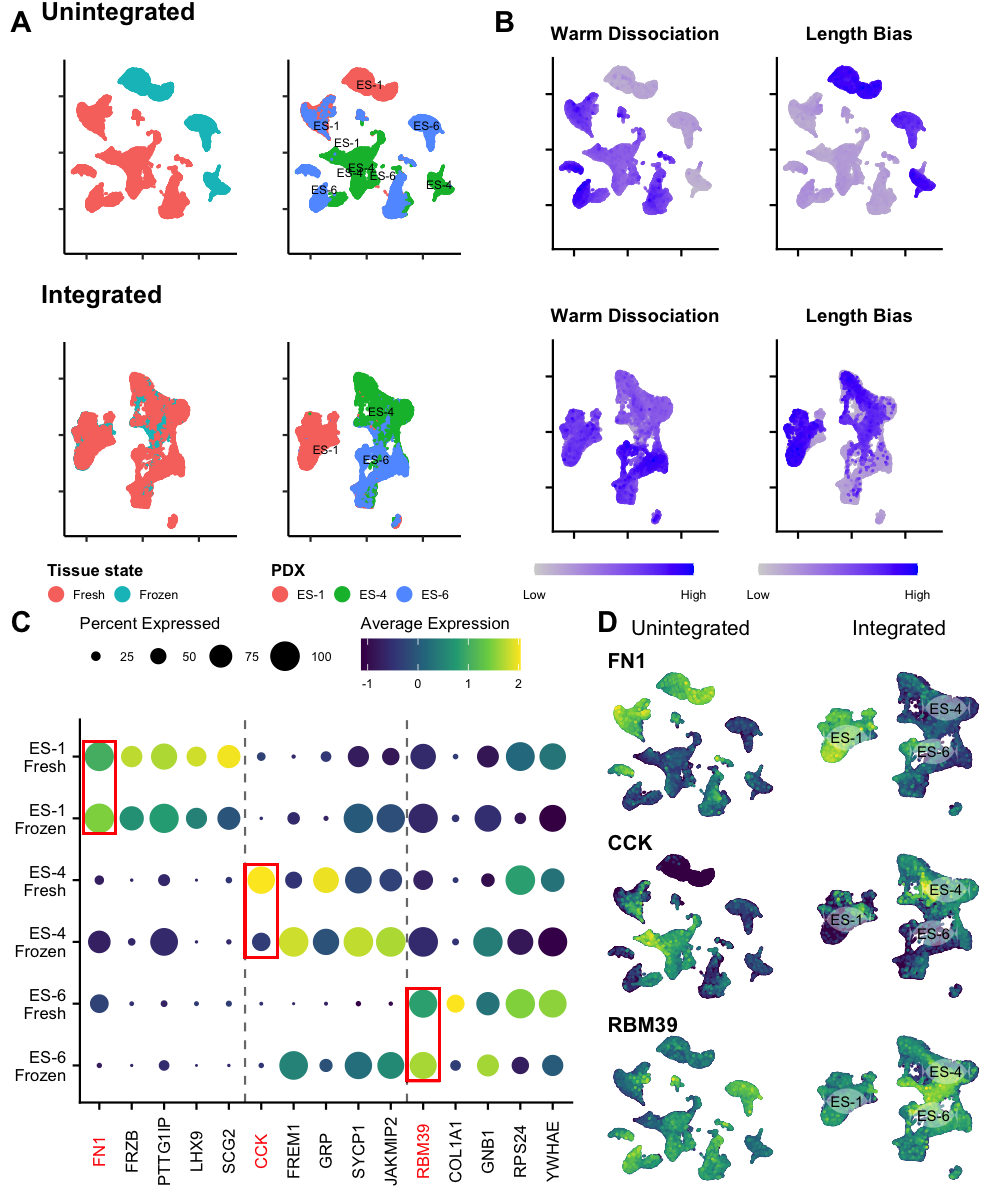


**Figure S9.** **Integration recovers matching cell states from different dissociation methods for ES. A** UMAP embeddings after integration (bottom row) showed alignment of matching PDX specimens. **B** After integration (bottom row), clusters on the UMAP were no longer affected by the identified biases. Darker blue indicates higher expression. **C** Dot plot of conserved markers shows unique gene expression for each PDX. The top marker is in red. **D** After integration (right column), top conserved markers are correctly aligned with the associated PDX cluster.


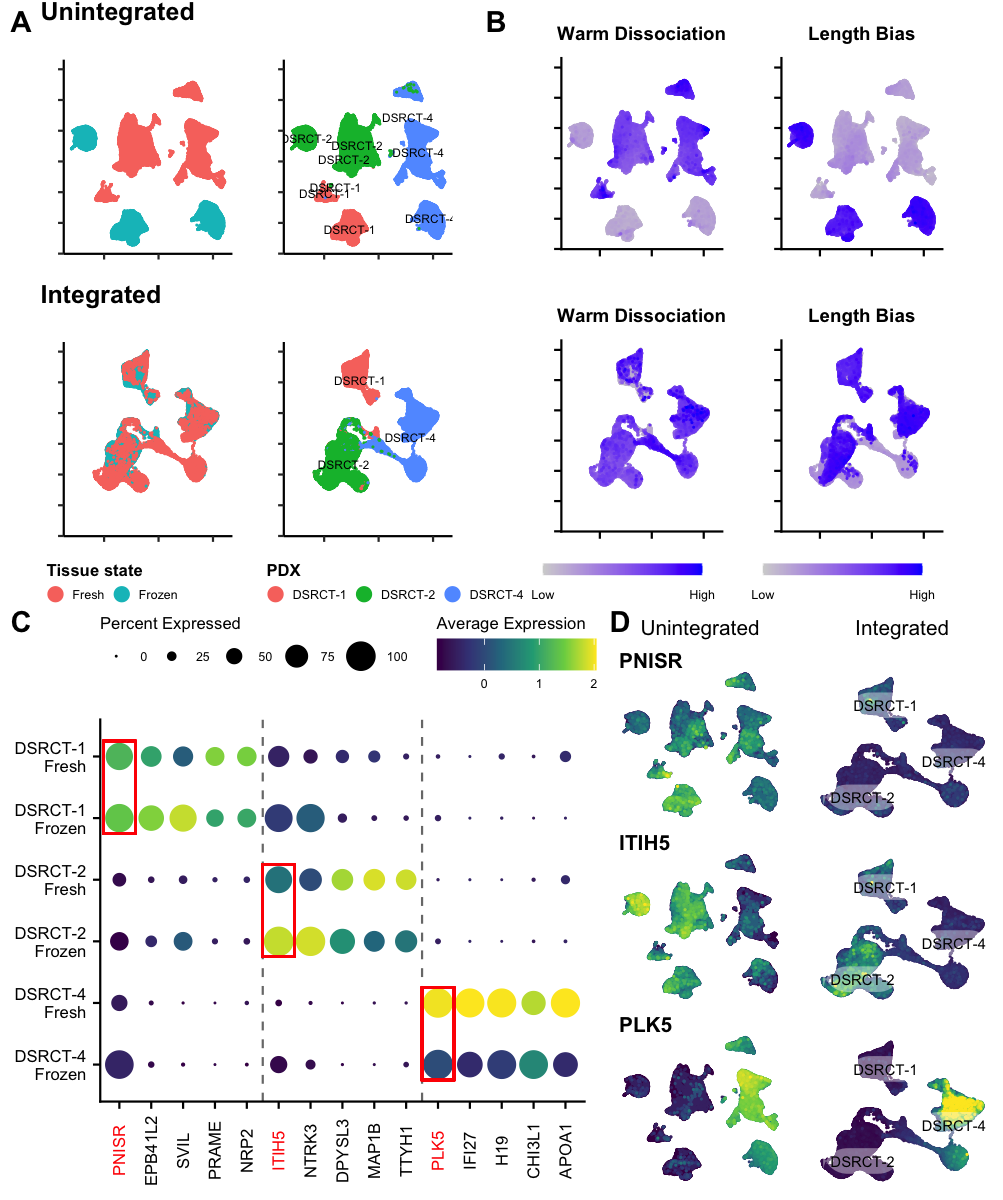


**Figure S10.** **Integration recovers matching cell states from different dissociation methods for DSRCT. A** UMAP embeddings after integration (bottom row) showed alignment of matching PDX specimens. **B** After integration (bottom row), clusters on the UMAP were no longer affected by the identified biases. Darker blue indicates higher expression. **C** Dot plot of conserved markers shows unique gene expression for each PDX. The top marker is in red. **D** After integration (right column), top conserved markers are correctly aligned with the associated PDX cluster.

**Additional Tables**

**Table S1.** Number of cells analyzed pre- and post-quality control.

**Table S2.** Gene sets curated from literature analyzed in PDX samples. The AddModuleScore function in Seurat v3 was used to observe the averaged gene expression of the gene sets.

**Table S3.** Differentially expressed genes between Warm and Nuclei protocols for ES. Wilcoxon test was used to compare gene expression between protocols.

**Table S4.** Differentially expressed genes between Warm and Nuclei protocols for OS. Wilcoxon test was used to compare gene expression between protocols.

**Table S5.** Differentially expressed genes between Warm and Nuclei protocols for DSRCT. Wilcoxon test was used to compare gene expression between protocols.

**Table S6.** Differentially expressed genes between Warm and Cold protocols for DSRCT. Wilcoxon test was used to compare gene expression between protocols.

**Table S7.** Differentially expressed genes between Warm and Cold protocols for ES. Wilcoxon test was used to compare gene expression between protocols.

**Table S8.** Top 200 longest genes with the most polyA regions. The polyA stretches were defined as greater than 15 A repeats within the full-length cDNA, including intronic and exonic regions for every gene.
